# Lessons From the Aftermaths of Green Revolution on Food System and Health

**DOI:** 10.3389/fsufs.2021.644559

**Published:** 2021-02-22

**Authors:** Daisy A. John, Giridhara R. Babu

**Affiliations:** 1 Public Health Foundation of India, Bangalore, India; 2 Head-Lifecourse Epidemiology, Public Health Foundation of India, Bangalore, India

**Keywords:** green revolution, sustainability, food system, agriculture, India

## Abstract

Food production has seen various advancements globally in developing countries, such as India. One such advancement was the green revolution. Notably, the World Bank applauds the introduction of the green revolution as it reduced the rural poverty in India for a certain time. Despite the success of the green revolution, the World Bank reported that health outcomes have not been improved. During the post-green revolution period, several notable negative impacts arose. Exclusive studies were not conducted on the benefits and harms before the introduction of the green revolution. Some of such interventions deviate from the natural laws of balance and functioning and are unsustainable practices. To avoid the adverse effects of some of these developments, a review of these interventions is necessary.

## Introduction

The production of food within India was insufficient in the years from 1947 to 1960 as there was a growing population, during which a famine was also anticipated (Nelson et al., 2019). Food availability was only 417 g per day per person (Ghosh, 2002). Many farmers were in debt, and they had become landless laborers. Political situations that prevailed also had a negative impact on the food system. There was a severe shortage of food crops as well as commercial crops. At the same time, Norman Borlaug, an agronomist, contributed to the green revolution significantly, and this had set out its effects throughout the world. He provided new seeds for cultivation, which were stocky, disease-resistant, fast-growing, and highly responsive to fertilizers. In India, the green revolution was launched under the guidance of geneticist Dr. M. S. Swaminathan (Somvanshi et al., 2020). It started around 1960s and helped in increasing food production in the country. The green revolution’s primary aim was to introduce high-yielding varieties (HYVs) of cereals to alleviate poverty and malnutrition (Nelson et al., 2019). Not to deny, the green revolution was capable of mitigating hunger and malnutrition in the short term as well (Davis et al., 2019).

### What Is the Green Revolution?

The green revolution led to high productivity of crops through adapted measures, such as (1) increased area under farming, (2) double-cropping, which includes planting two crops rather than one, annually, (3) adoption of HYV of seeds, (4) highly increased use of inorganic fertilizers and pesticides, (5) improved irrigation facilities, and (6) improved farm implements and crop protection measures (Singh, 2000; Brainerd and Menon, 2014) and modifications in farm equipment. There was a high investment in crop research, infrastructure, market development, and appropriate policy support (Pingali, 2012). Efforts were made to improve the genetic component of traditional crops. This included selection for higher yield potential; wide adaptation to diverse environments; short growth duration; superior grain quality; resistance to biotic stress, insects, and pests; and resistance to abiotic stress, including drought and flooding (Khush, 2001). After the green revolution, the production of cereal crops tripled with only a 30% increase in the land area cultivated. This came true all over the world, with a few exceptions. In addition, there were significant impacts on poverty reduction and lower food prices. Studies also showed that without the green revolution, caloric availability would have declined by around 11–13%. These efforts benefitted all consumers in the world, particularly the poor. There were further improved returns to the crop improvement research. It also prevented the conversion of thousands of hectares of land for agriculture (Pingali, 2012). The green revolution helped India move from a state of importing grains to a state of self-sufficiency (Brainerd and Menon, 2014). Earlier, it was the ship-to-mouth system, i.e., India depended on imported food items (Ramachandran and Kalaivani, 2018). There are undoubtedly positive effects on the overall food security in India. Correspondingly, useful and elaborate evidence in support of the positive impact of the green revolution is available. However, after a certain period, some unintended but adverse effects of the green revolution were noticed. This paper introspects the negative impacts of the green revolution on the food system in India. Studies by the departments of conventional agriculture, social sector development, etc. bring out the positive impacts of the green revolution, such as increased yield and reduced mortality and malnutrition (Somvanshi et al., 2020; von der Goltz et al., 2020). On the other hand, studies conducted by the environmental and public health departments suggest that to mitigate the negative impacts, a reduced usage of pesticides is sufficient (Gerage et al., 2017). There are many studies being conducted to find out the extent of the impacts of pesticides and insecticides and other similar chemicals.

Although there are many studies that focused on this topic, this paper makes an effort to inform policy by asserting that many interventions, beneficial for the shorter term, such as the green revolution, without the consideration of ecological principles, can be detrimental and irreversible in the long run (Clasen et al., 2019). Efforts to recover from environmental damage would require extensive efforts, time, and other resources as compared with the destruction of the environment. Hence, any new intervention needs to be checked for its eco-friendliness and sustainability features.

Carrying forward intensified usage of pesticides is not advisable in an ever-deteriorating environment, and alternative solutions that can promote economic growth, increased yield, and less harm to the environment can be implemented. The vicious cycle of problem-solution-negative impacts has to be broken at some point of time. For example, a second green revolution is focused on in various countries (Ameen and Raza, 2017; Armanda et al., 2019). Instead of this, techniques to promote sustainable agriculture can be considered. Hence, there has to be a wake-up call before the repetition of history.

### Impacts of the Green Revolution

#### Impacts on Agriculture and Environment

##### Pests and Pesticide

There has been a significant increase in the usage of pesticides, and India became one of the largest producers of pesticides in the whole of Asia (Narayanan et al., 2016). Although this has contributed to a lot of economic gains (Gollin et al., 2018), it is found out that a significant amount of pesticides is unnecessary in both industrialized and developing countries. For instance, it is reported that the presence of pesticides within freshwater is a costly concern with detected levels exceeding the set limits of pesticide presence (Choudhary et al., 2018). Although the average amount of pesticide usage is far lower than in many other countries, there is high pesticide residue in India. This causes a large amount of water pollution and damage to the soil. Another major issue is the pest attack, which arises due to an imbalance in the pests. Due to increased pesticide usage, the predator and prey pests are not in balance, and hence there is an overpopulation of one kind of pest that would attack certain crops. This leads to an imbalance in the production of those kinds of crops. These crops would need stronger pesticides or pesticides of new kinds to tackle the pests attacking those. This also has led to the disruption in the food chain (Narayanan et al., 2016).

##### Water Consumption

India has the highest demand for freshwater usage globally, and 91% of water is used in the agricultural sector now (Kayatz et al., 2019). Currently, many parts of India are experiencing water stress due to irrigated agriculture (Davis et al., 2018). The crops introduced during the green revolution were water-intensive crops. Most of these crops are cereals, and almost 50% of dietary water footprint is constituted by cereals in India (Kayatz et al., 2019). Since the crop cycle is less, the net water consumed by these crops is also really high. The production of rice currently needs flooding of water for its growth^1^ (International Rice Research Institute). Canal systems were introduced, and there were irrigation pumps that sucked out water from the groundwater table to supply the water-intensive crops, such as sugarcane and rice (Taylor, 2019). Punjab is a major wheat- and rice-cultivating area, and hence it is one of the highest water depleted regions in India^2^ (Alisjahbana, 2020). It is predicted that Punjab will have water scarcity in a few years (Kumar et al., 2018). Diminishing water resources and soil toxicity increased the pollution of underground water. The only aim of the green revolution was to increase food items’ production and make it sufficient to feed everyone. The environmental impacts were not taken into account (Taylor, 2019). Based on the previous allocation of budget, irrigation was allotted 9,828 crore INR as compared with 3,080 crore INR for agriculture, excluding irrigation. This pattern has been persistent in the past 3 years (NABARD, 2020). Overall, the GDP from agriculture is 380,239 crore INR (16.5% of GDP) (Economics, 2020; India, 2020). This indicates that there has been a higher investment on irrigation of water due to its increased need in comparison with the other inputs required for agriculture.

##### Air Pollution

Air pollution introduced due to the burning of agricultural waste is a big issue these days. In the heartland of the green revolution, Punjab, farmers are burning their land for sowing the crops for the next cycle instead of the traditionally practiced natural cycle. The next crop cycle arrives very soon because the crop cycle is of short duration for the hybrid crops introduced in the green revolution. This contributes to the high amount of pollution due to the burning of agricultural waste in parts of Punjab (Davis et al., 2018). This kind of cultivation can lead to the release of many greenhouse gases, such as carbon dioxide, methane, nitrogen oxides, etc. (de Miranda et al., 2015).

##### Impacts on Soil and Crop Production

There was a repetition of the crop cycle for increased crop production and reduced crop failure, which depleted the soil’s nutrients (Srivastava et al., 2020). Similarly, as there is no return of crop residues and organic matter to the soil, intensive cropping systems resulted in the loss of soil organic matter (Singh and Benbi, 2016). To meet the needs of new kinds of seeds, farmers used increasing fertilizers as and when the soil quality deteriorated (Chhabra, 2020). The application of pesticides and fertilizers led to an increase in the level of heavy metals, especially Cd (cadmium), Pb (lead), and As (arsenic), in the soil. Weedicides and herbicides also harm the environment. The soil pH increased after the green revolution due to the usage of these alkaline chemicals (Sharma and Singhvi, 2017). The practice of monoculture (only wheat–rice cultivation) has a deleterious effect on many soil properties, which includes migration of silt from the surface to subsurface layers and a decrease in organic carbon content (Singh and Benbi, 2016). Toxic chemicals in the soil destroyed beneficial pathogens, which are essential for maintaining soil fertility. There is a decrease in the yield due to a decline in the fertility of the soil. In addition, the usage of tractors and mechanization damaged the physicochemical properties of the soil, which affected the biological activities in the soil. In the traditional methods, soil recovers in the presence of any kind of stressors (Srivastava et al., 2020). However, this does not happen with these modern methods. In a study conducted in Haryana, soil was found to have waterlogging, salinity, soil erosion, decline, and rise of groundwater table linked to brackish water and alkalinity, affecting production and food security in the future (Singh, 2000).

Although for around 30 years there was an increase in the production of crops, the rice yield became stagnant and further dropped to 1.13% in the period from 1995 to 1996 (Jain, 2018). Similarly with wheat, production declined from the 1950s due to the decrease in its genetic potential and monoculture cropping pattern (Handral et al., 2017). The productivity of potato, cotton, and sugarcane also became stagnant (Singh, 2008). Globally, agriculture is on an unsustainable track and has a high ecological footprint now (Prasad, 2016).

##### Extinction of Indigenous Varieties of Crops

Due to the green revolution, India lost almost 1 lakh varieties of indigenous rice (Prasad, 2016).

**Figure 1:**
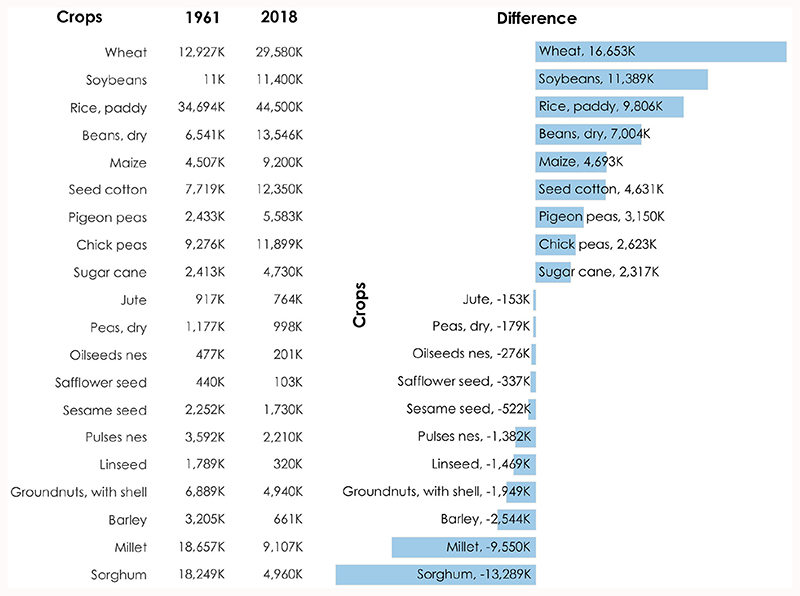
Changes in area harvested of the crops from the years 1961 to 2018 (data source: FAOSTAT; FAO, 2020).

Since the time of the green revolution, there was reduced cultivation of indigenous varieties of rice, millets, lentils, etc. In turn, there was increased harvest of hybrid crops, which would grow faster (Taylor, 2019). This is indicated in Figure 1. There is a large increase in the cultivation of wheat, soybeans, and rice. In addition, there is a large decrease in the cultivation of sorghum, other millets, barley, and groundnuts. The increase in certain crops was due to the availability of HYVs of seeds and an increase in the area of production of these crops (Singh, 2019). The preference of farmers also changed in terms of the cultivation of crops. The native pulses, such as moong, gram, tur, etc., and some other oilseed crops, such as mustard, sesame, etc., were not cultivated further on a larger scale than it was before. Traditionally grown and consumed crops, such as millets, grow easily in arid and semi-arid conditions because they have low water requirements. However, there was the unavailability of high-yielding seeds of millets, and hence farmers moved to only rice and wheat (Srivastava et al., 2020).

#### Impacts on Human Health

##### Food Consumption Pattern

Traditionally, Indians consumed a lot of millets, but this became mostly fodder after the green revolution (Nelson et al., 2019). The Cambridge world history of food mentions that the Asian diet had food items, such as millets and barley (Kiple and Ornelas, 2000). As already mentioned, after the period of the green revolution, there were significant changes in food production, which in turn affected the consumption practices of Indians. The Food and Agriculture Organization (FAO) has recorded that over the years 1961–2017, there are a decrease in the production of millets and an increase in the production of rice (Food and Agricultural Organisation, 2019; Smith et al., 2019); thus, rice became the staple diet of the country. Though the green revolution made food available to many, it failed to provide a diverse diet but provided increased calorie consumption.

##### Health-Related Impacts on the General Population

Most of the pesticides used belong to the class organophosphate, organochlorine, carbamate, and pyrethroid. Indiscriminate pesticide usage has led to several health effects in human beings in the nervous, endocrine, reproductive, and immune systems. Sometimes, the amount of pesticide in the human body increases beyond the capacity of the detoxification system due to continuous exposure through various sources (Xavier et al., 2004). Of all, the intake of food items with pesticide content is found to have high exposure, i.e., 10^3^-10^5^ times higher than that arising from contaminated drinking water or air (Sharma and Singhvi, 2017).

##### Impacts on Farmers

Most of the farmers who use pesticides do not use personal protective gear, such as safety masks, gloves, etc., as there is no awareness about the deleterious effects of pesticides. Pesticides, applied over the plants, can directly enter the human body, and the concentration of nitrate in the blood can immobilize hemoglobin in the blood. Organophosphates can also develop cancer if exposed for a longer period. Since it is in small quantities, the content may not be seen or tasted; however, continuous use for several years will cause deposition in the body. Dichlorodiphenyltrichloroethane (DDT) was a very common pesticide used in India, now banned internationally as it is found to bioaccumulate and cause severe harmful effects on human beings (Sharma and Singhvi, 2017). However, there is still illegal use of DDT in India. In India, women are at the forefront of around 50% of the agricultural force. Hence, most of these women are directly exposed to these toxins at a young age and are highly vulnerable to the negative impacts including effects on their children. It is proven that there is a significant correlation between agrochemical content in water and total birth defects. The damaging impact of agrochemicals in water is more pronounced in poor countries, such as India (Brainerd and Menon, 2014).

## Discussion

Efforts are underway to produce genetic variants of millets that can withstand biotic and abiotic stresses. Earlier, the introduction of genetic variants of rice and wheat and pesticides was the solution for malnutrition, but it led to environmental destruction in a few years. In the short term, food scarcity might rise again due to increased water depletion and soil damage. Any new interventions should be carefully introduced not to disrupt other systems to prevent future adversities. A domino effect is expected to occur when there is any disruption in the ecosystem, such that if even one link in the food chain is affected, it affects other parts of the chain also. Most of the ecological disruption is by human intervention (Vaz et al., 2005). Pesticides used for agricultural activities are released to the environment through air drift, leaching, and run-off and are found in soil, surface, and groundwater. This can contaminate soil, water, and other vegetation. Pesticide residues are found to be present in almost all habitats and are detected in both marine and terrestrial animals (Choudhary et al., 2018). The mechanisms include absorption through the gills or teguments, which is bioconcentration, as well as through the consumption of contaminated food, called biomagnification or bioamplification. In marine systems, seagrass beds and coral reefs were found to have very high concentrations of persistent organic pollutants (Dromard et al., 2018). It also affects the activities of insects and microbes. It kills insects and weeds, is toxic to other organisms, such as birds and fish, and contaminates meat products, such as chicken, goat, and beef. This can lead to bioaccumulation in human beings along with poor food safety, thus impairing nutrition and health. Repeated application leads to loss of biodiversity (Choudhary et al., 2018). Consumption of pesticide-laden food can lead to loss of appetite, vomiting, weakness, abdominal cramps, etc. (Gerage et al., 2017). There is a decline in the number of pollinators, for instance, the destruction of bumblebee colonies that are an important group of pollinators on a global scale (Baron et al., 2017). There is an extinction of honeybee populations, and it poses a great threat to the survival of human beings (Hagopian, 2017). The residue level of these pesticides depends on the organism’s habitat and position in the food chain. This is a serious issue because the predicted usage of pesticides is that it will be doubled in the coming years (Choudhary et al., 2018).

In addition, it is not nearly possible to get back the lost varieties of indigenous rice. Likewise, further advancements should not lead to the extinction of the other indigenous varieties of grains, such as millets.

In conclusion, the effects of the green revolution are persisting. The green revolution, which was beneficial in ensuring food security, has unintended but harmful consequences on agriculture and human health. This requires new interventions to be tested and piloted before implementation, and continuous evaluation of the harms and benefits should guide the implementation. An already fragile food system is affected due to the aftermaths of the green revolution. The potential negative impacts are not part of the discourse as it can affect the narratives of development and prosperity. Developments introduced due to necessity may not be sustainable in the future. Organic ways of farming need to be adopted for sustainable agricultural practices. Similarly, alternative agriculture techniques, such as intercropping, Zero Budget Natural Farming (ZBNF) with essential principles involving the enhancement of nature’s processes, and elimination of external inputs, can be practiced (Khadse et al., 2018). The government of Andhra Pradesh (AP), a Southern state in India, has plans to convert 6 million farmers and 8 million hectares of land under the state initiative of Climate Resilient Zero Budget Natural Farming because of the positive outputs obtained in the ZBNF impact assessments in the states of Karnataka and AP (Reddy et al., 2019; Koner and Laha, 2020) In AP, it was observed that yield of crops increased to 9% in the case of paddy and 40% in the case of ragi. Net income increased from 25% in the case of ragi ranging to 135% in the case of groundnut (Martin-Guay et al., 2018; Reddy et al., 2019). There is a need for a systems approach in dealing with food insecurity and malnutrition and other similar issues. Like the already mentioned example, the green revolution was brought in to reduce the problem of reduced yield. Now, there is a green revolution 2 that is planned. Before such interventions are taken, environmental risk assessments and other evaluation studies should be conducted for a sustainable future.
